# Effect of extracorporeal shock wave for tennis elbow: A protocol for systematic review of randomized controlled trial

**DOI:** 10.1097/MD.0000000000014517

**Published:** 2019-02-15

**Authors:** Hua-yu Tang, Tao Yu, Wei Wei, Yu Zhao

**Affiliations:** a Second Ward of Orthopedics Department, First Affiliated Hospital of Jiamusi University, Jiamusi; b Department of Orthopedics, Huludao Central Hospital, Huludao, China.

**Keywords:** effectiveness, extracorporeal shock wave, safety, stroke, systematic review, tennis elbow

## Abstract

**Background:**

Previous clinical studies have reported that extracorporeal shock wave (EPSW) is an effective treatment for patients with tennis elbow (TE). However, no systematic review has assessed its effectiveness and safety for the treatment of TE.

**Methods:**

In this systematic review, we will search the potential eligible literature from the following electronic databases: Central, Embase, MEDLINE, CINAHL, and CNKI from inception to the present. All literatures of randomized controlled trials of EPSW for TE will be considered without language restrictions. Two reviewers will independently select the studies, extract the data, and evaluate the methodology quality. All disagreements between those 2 reviewers will be resolved by a third reviewer involved through discussion. Outcome data will be pooled by RevMan 5.3 software if the heterogeneity is reasonable. Reporting bias will also be conducted if more than 10 included studies can be reached.

**Results:**

This systematic review will evaluate the clinical effectiveness and safety of EPSW for TE.

**Conclusion:**

The findings of this study will summarize the current evidence of EPSW on TE outcomes and may provide guidance for both clinical practice and further studies.

**Dissemination and ethics:**

This systematic review does not need ethical approval, because it does not utilize the individual patient data. Its findings are expected to publish in peer-reviewed journals.

**Systematic review registration:**

PROSPERO CRD42019119687.

## 1 Introduction

Tennis elbow (TE) is a common condition among general population.^[1–3]^ It often manifests as elbow pain, swelling or tenderness.^[4–6]^ It has been reported that its incidence is about 4 to 7 subjects per 1000 people annually.^[7–9]^ Further study reported that about 1% to 3% subjects experience this disorder over the course of their lifetime, and mostly affects individuals between 35 and 50 years old.^[10]^ If this condition cannot be managed timely and effectively, it can greatly affect the quality of life in subjects with TE.

Several managements are used to treat this condition, including medications, activity modification, physical therapy, as well as the local corticosteroid injection.^[11–14]^ However, many subjects still report a low recovery rate achieved after the treatment.^[11,12]^ Therefore, additional alternative therapies are still needed to treat this condition.

Fortunately, a variety of studies reported that alternative therapy, such as acupuncture, extracorporeal shock wave (EPSW) is effective for the management of TE, especially for EPSW.^[15–20]^ However, no systematic review has been conducted to assess the effectiveness and safety of EPSW for patients with TE. Thus, the protocol of present systematic review and meta-analysis will focus on evaluating the effectiveness and safety of EPSW for TE.

## 2 Methods

### 2.1 Objectives

The aim of this systematic review is to assess the effectiveness and safety of EPSW for TE.

### 2.2 Study registration

The protocol of this systematic review has been registered on http://www.crd.york.ac.uk/PROSPERO with CRD42019119687. It is designed and reported based on the Preferred Reporting Items for Systematic Reviews and Meta-Analysis Protocol statement guidelines.^[21]^

### 2.3 Inclusion criteria for study selection

#### 2.3.1 Type of study

Only randomized controlled trial (RCT) of EPSW for TE will be considered for inclusion. Other studies, such as non-RCT, quasi-RCT, case–control study, case reports, case series, and nonclinical studies will not be considered.

#### 2.3.2 Type of participants

Participants clinically diagnosed of TE will be included regardless of their gender, age, and race. However, patients will be excluded if TE resulted from other disorders, such as previous elbow surgery, and trauma.

#### 2.3.3 Type of interventions

Any RCTs for assessing the effectiveness and safety of EPSW for TE will be included. However, the studies involved the combination of EPSW with other treatments will not be considered. The control treatments will consist of any other interventions that will not include any types of EPSW intervention.

#### 2.3.4 Type of outcome measurements

Primary outcome includes elbow pain intensity. It will be measured by any pain measurement tools, such as visual analog scale or numeric Rating Scale, and any others. Secondary outcomes consist of elbow function, as assessed by related scales, including Mayo Elbow Performance Score, American Shoulder and Elbow Surgeons Elbow scores, and abbreviated Disability of the Shoulder and Hand score; quality of life, as evaluated by 36-Item Short Form Health Survey, and any other associated scales; as well as any adverse events.

### 2.4 Search methods for the identification of studies

#### 2.4.1 Electronic searches

The following databases will be retrieved from inception to the present, including Cochrane Central Register of Controlled Trials, Embase, MEDLINE, Cumulative Index to Nursing and Allied Health Literature, and China National Knowledge Infrastructure. The details of search strategy for MEDLINE are shown in Table 1. The equivalent strategies will be applied for other databases, and will be also translated into Chinese while searching the Chinese database.

**Table 1:**
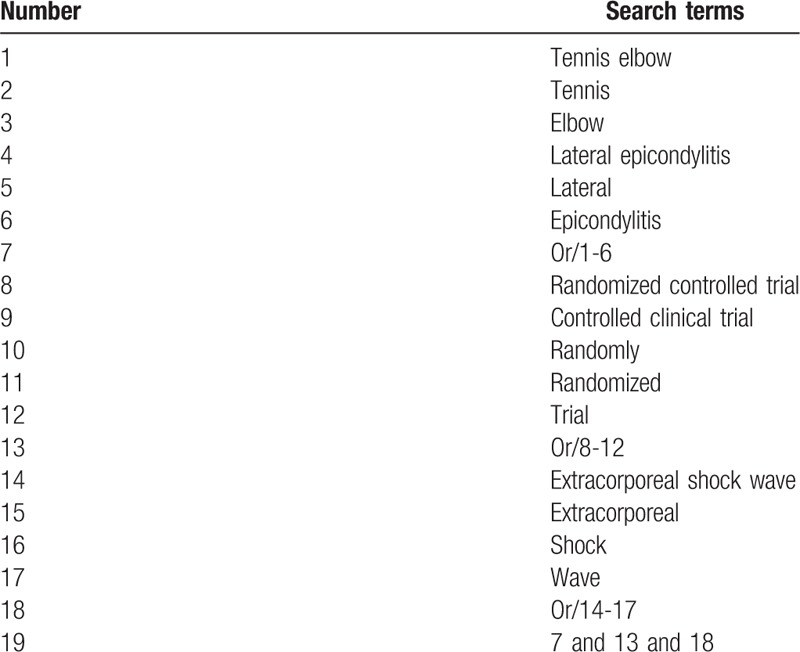
Search strategy applied in MEDLINE database.

#### 2.4.2 Search for other resources

Additionally, website of clinical registry, dissertations, as well as the reference lists of all included studies will also be searched to avoid missing any potential eligible studies.

### 2.5 Data collection and analysis

#### 2.5.1 Study selection

**Figure 1:**
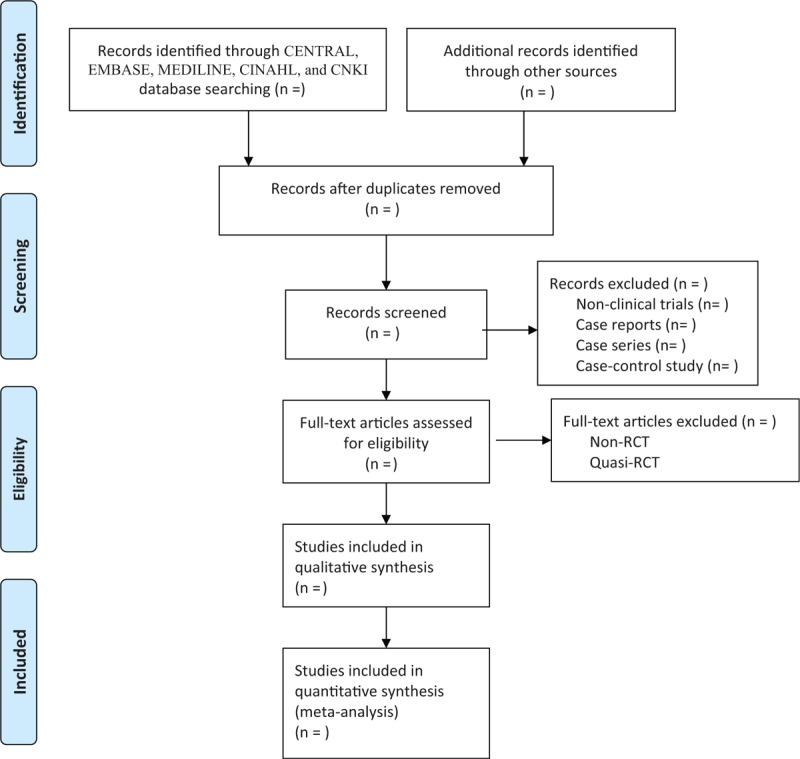
Process of study selection.

Two reviewers will independently screen the titles, abstracts, as well as full texts according to the predefined criteria. All select procedures will be performed based on the PRISMA flowchart. Any oppositions regarding the study selection occurred between 2 reviewers will be resolved by a third reviewer through discussion. The process of study selection is shown in Figure 1.

#### 2.5.2 Data extraction and management

Two reviewers will also independently extract data from each included study by using a predefined standard data extraction form. It includes detailed information of title, first author, published data, region, study design, study methods, such as the procedures of randomization, allocation, and blinding, outcome measurements, and any other details. Any divergences will be handled by a third reviewer through discussion.

#### 2.5.3 Risk of bias assessment

Cochrane tool of risk of bias will be utilized to evaluate the risk of bias for each included study by 2 independent reviewers. Each study will be assessed on 7 domains, and each item will be presented as a high risk of bias, unclear risk of bias, or low risk of bias. Any differences of risk of bias evaluation will be dealt with a third reviewer through discussion.

#### 2.5.4 Measurement of treatment effect

The continuous data will be presented as the mean difference, or standardized mean difference with 95% confidence intervals (CIs). The dichotomous data will be presented as the risk ratio with 95% CIs. If ordinal data are available only, they will be converted to dichotomous data.

#### 2.5.5 Missing data management

For any insufficient, unclear or even missing data, we will contact the original corresponding authors to request those data. If those additional data cannot be achieved, the current available data will be pooled and analyzed, and it will be discussed in Section 3.

#### 2.5.6 Assessment of heterogeneity

The tests of *I*^*2*^ and *χ*^*2*^ will be utilized to detect the heterogeneity. A fair and reasonable heterogeneity is defined as the value of *I*^*2*^ ≤ 50%. Otherwise, it will be regarded as having substantial heterogeneity.

#### 2.5.7 Data synthesis

If the *I*^*2*^ ≤ 50%, the outcome data will be pooled by using fixed-effect model, and a meta-analysis will be carried out. Otherwise, the random-effect model will be used. Meanwhile, subgroup analysis will be conducted to identify any possible factors that may cause such situation. If the heterogeneity is still significant post the subgroup analysis, the data will not be pooled and a meta-analysis will not be performed. However, a narrative summary will be presented instead.

#### 2.5.8 Subgroup analysis

If substantial heterogeneity is identified, subgroup analysis will be conducted based on the different forms of treatments, controls, and outcomes.

#### 2.5.9 Sensitivity analysis

If it is possible, we will also conduct sensitivity analysis to check the robustness of the pooled results according to the methodological qualities, and statistical models.

#### 2.5.10 Publication biases

If more than 10 eligible studies will be included, funnel plot will be performed to detect the potential publication biases.^[22]^ In addition, Egg's and Begg's tests will be conducted to identity that if the funnel plot is asymmetry.^[23]^

## 3 Discussion

TE is gravely tormenting individuals and greatly reduces the quality of life in patients who experience it. EPSW is a nonpharmaceutical therapy that is reported to treat TE effectively. However, no systematic review has been conducted to assess the effectiveness and safety of EPSW for the treatment of TE. Thus, this systematic review will firstly explore this issue.

In the current systematic review, we will retrieve all associated and potential eligible studies without language restrictions. All studies related to the EPSW for the treatment of TE will be fully considered. The results of this systematic review will present a summary of the updated evidence on the effectiveness and safety of EPSW for patients with TE. It may also provide helpful evidence for the clinical practice and even the health policy-makers.

## Author contributions

**Conceptualization:** Tao Yu, Yu Zhao.

**Data curation:** Hua-yu Tang, Tao Yu, Wei Wei, Yu Zhao.

**Formal analysis:** Tao Yu, Wei Wei.

**Investigation:** Hua-yu Tang.

**Methodology:** Tao Yu, Wei Wei, Yu Zhao.

**Project administration:** Hua-yu Tang.

**Resources:** Tao Yu, Wei Wei, Yu Zhao.

**Software:** Tao Yu, Wei Wei.

**Supervision:** Hua-yu Tang.

**Validation:** Hua-yu Tang, Tao Yu, Yu Zhao.

**Visualization:** Hua-yu Tang, Tao Yu, Yu Zhao.

**Writing – original draft:** Hua-yu Tang, Tao Yu, Wei Wei, Yu Zhao.

**Writing – review & editing:** Hua-yu Tang, Wei Wei, Yu Zhao.
